# “Healthy immigrant effect” among individuals experiencing homelessness in Spain?: Foreign-born individuals had higher average age at death in 15-year retrospective cohort study

**DOI:** 10.1186/s12889-023-16109-5

**Published:** 2023-06-22

**Authors:** Fran Calvo, Ana Guillén, Xavier Carbonell, Rebeca Alfranca, Marta Beranuy, Alícia Parés-Bayerri, Sílvia Font-Mayolas

**Affiliations:** 1 Serra Húnter Fellow, Department of Pedagogy, Quality of Life Research Institute, Universitat de Girona, Girona, Spain; 2 Department of Personality, Evaluation and Clinical Psychology, Universidad Complutense, Madrid, Spain; 3 FPCEE, Universitat Ramon Llull, Blanquerna, Barcelona, Spain; 4 Primary Care Centre Santa Clara, Catalan Institute of Health, Girona, Spain; 5 Department of Health Sciences, Faculty of Health Sciences, Universidad Pública de Navarra (UPNA), Pamplona, Spain; 6 Cyberpsychology research group, Universidad Internacional de La Rioja, Logroño, Spain; 7 Department of Psychology, Quality of Life Research Institute, Universitat de Girona, Girona, Spain

**Keywords:** Homelessness, Immigration, Mortality, Health, Immigrant advantage, Substance use, Substance use disorder, COPD

## Abstract

**Background:**

Individuals experiencing homelessness (IEHs) suffer from severe health inequities. Place of origin is linked to health and mortality of IEHs. In the general population the “healthy immigrant effect” provides a health advantage to foreign-born people. This phenomenon has not been sufficiently studied among the IEH population. The objectives are to study morbidity, mortality, and age at death among IEHs in Spain, paying special attention to their origin (Spanish-born or foreign-born) and to examine correlates and predictors of age at death.

**Methods:**

Retrospective cohort study (observational study) of a 15-year period (2006–2020). We included 391 IEHs who had been attended at one of the city’s public mental health, substance use disorder, primary health, or specialized social services. Subsequently, we noted which subjects died during the study period and analyzed the variables related to their age at death. We compared the results based on origin (Spanish-born vs. foreign-born) and fitted a multiple linear regression model to the data to establish predictors of an earlier age at death.

**Results:**

The mean age at death was 52.38 years. Spanish-born IEHs died on average almost nine years younger. The leading causes of death overall were suicide and drug-related disorders (cirrhosis, overdose, and chronic obstructive pulmonary disease [COPD]). The results of the linear regression showed that earlier death was linked to COPD (b = − 0.348), being Spanish-born (b = 0.324), substance use disorder [cocaine (b =-0.169), opiates (b =.-243), and alcohol (b =-0.199)], cardiovascular diseases (b = − 0.223), tuberculosis (b = − 0.163), high blood pressure (b =-0.203), criminal record (b =-0.167), and hepatitis C (b =-0.129). When we separated the causes of death for Spanish-born and foreign-born subjects, we found that the main predictors of death among Spanish-born IEHs were opiate use disorder (b =-0.675), COPD (b =-0.479), cocaine use disorder (b =-0.208), high blood pressure (b =-0.358), multiple drug use disorder (b =-0.365), cardiovascular disease (b =-0.306), dual pathology (b =-0.286), female gender (b =-0.181), personality disorder (b =-0.201), obesity (b =-0.123), tuberculosis (b =-0.120) and having a criminal record (b =-0.153). In contrast, the predictors of death among foreign-born IEHs were psychotic disorder (b =-0.134), tuberculosis (b =-0.132), and opiate (b =-0.119) or alcohol use disorder (b =-0.098).

**Conclusions:**

IEHs die younger than the general population, often due to suicide and drug use. The healthy immigrant effect seems to hold in IEHs as well as in the general population.

Individuals experiencing homelessness (IEHs) suffer from severe inequities in a wide range of biological, psychological, and social conditions [1]. As a result, IEHs have a lower life expectancy [2]. One of the most accepted explanations for the high mortality of IEHs is that they are subject to an accumulation of risk factors such as drug use (including tobacco and alcohol) and mental health problems [3]. In addition, IEHs are particularly vulnerable to death from injury, overdose, and other external causes [4, 5], and from cardiovascular and respiratory conditions [1]. Moreover, they are more likely to suffer from infections (especially hepatitis and tuberculosis). Treatment of active tuberculosis in IEHs can be complicated by nonadherence to therapy, prolonged infectivity, and the development of drug resistance [6]. Human immunodeficiency virus (HIV) infection is also more prevalent in this population. Common risk factors for HIV infection among IEHs include prostitution, multiple sexual partners, inconsistent use of condoms, and injection drug use [7].

There are subgroups of IEHs whose specific characteristics imply differentiated risk factors and causes of mortality. The death of young IEHs is associated with external causes, especially suicide and overdose [8], while death in adult IEHs is associated with diseases typical of the general population. However, the age at death among IEH adults is between 10 and 15 years lower than that of the general adult population, according to the study by Baggett et al. [2] or 20 years lower, according to that by Termorshuizen et al. [9].

Another specific population to consider when establishing differences in health, especially in mental health, is the foreign-born population. Studies on foreign-born IEHs have reported a health advantage for immigrants experiencing homelessness compared to people born in the country [10–12]. This advantage has to do with greater youth, a better state of general health (since, given the difficulties of migration, people who migrate tend to be healthier than the general population in their home country) and that the causes of homelessness differ from that of locally born populations and are more related to poverty and migration [13]. Foreign-born people—especially recent immigrants—are less likely to suffer from chronic health problems, mental health problems and/or substance use disorder [14].

Studies carried out with the general population show that migration increases the risk of homelessness and has a negative impact on health. In the general population, worse mental health has been reported in people who have migrated, as a result of the associated stress [15]. In contrast, migration is associated with better health during the first ten years of residence in the receiving country compared to locally born people [16]. Studies carried out in Canada show a higher age at death among foreign-born people compared to those born in the country. This advantage is less clear for morbidity, especially if migration occurs during adulthood and not in the perinatal period, childhood/adolescence or at the end of life [17, 18]. In Europe, there are North-South differences in the health status of immigrants compared to those born in the receiving country, which are partly explained by the poorer economic and social conditions of immigrants in some countries. Their state of health is better in Italy and Spain than in France and Belgium, where there are greater difficulties surrounding integration [19].

In Spain, the differences in the health of foreign-born individuals with respect to Spanish-born individuals depend on socioeconomic factors such as poverty and socioeconomic status. Gotsens et al. found that foreign-born born people who arrived before 2006 had worse self-rated health than locally born people, despite the fact that the latter group had a higher prevalence of disability, illness, and mental disorders. On the other hand, foreign-born people who arrived after 2006 had better mental health than Spanish-born people. These advantages tended to be diluted by the effect of the economic crisis that began in 2008 [20]. The health advantages in foreign-born people can occur even a generation later, with children born in the receiving country to foreign-born parents experiencing better health than the children of locally born parents [21].

There is a gap in the scientific literature on the effect of migration on the health of IEHs. In Spain, we especially need studies to analyze this effect, as well as its relationship with health and mortality since in some areas the foreign-born IEH population represents more than half of the total IEH population [22]. Such a study in Spain is also important because, due to its geographical location, Spain is a point of entry for thousands of people who migrate to other European countries. The few studies carried out in Spain confirm an advantage in the health of immigrant IEHs [23], especially newcomers [10], but this phenomenon has still not been sufficiently studied. Thus, the objectives of this study were: (1) To study the mortality and age at death among IEHs in Spain; (2) To compare the age at death and its characteristics among people of IEHs in relation to their origin (Spanish-born or foreign-born); (3) To examine correlates and predictors of age at death.

## Method

### Design

A retrospective cohort study (observational study) of a 15-year period.

### Population under study

We included the 391 IEHs that died between 2006 and 2020 (both included) and that had been attended by one of the city of Girona (Spain)’s public mental health, substance use disorder, primary health, or specialized social services (day center, overnight shelter, or street team). All of the subjects met the criteria for literal homelessness, i.e., people living in public space or whose overnight housing situation forces them to spend the day on the streets or in situations of extreme substandard housing (European Homelessness Observatory categories 1, 2, 3d, 3e, 3f and 3 h of homelessness and residential exclusion) (Busch-Geertsema et al., 2016). These categories refer to: (i) people without accommodation, sleeping on the streets, in public space, in vehicles or under some form of makeshift cover; (ii) persons living in any type of temporary or crisis accommodation; (iii) persons illegally occupying conventional housing; (iv) persons living in conventional dwellings unfit for human habitation; (v) persons living in trailers, tents or caravans; and (vi) persons living in non-conventional buildings and temporary structures or settlements.

### Procedure

Some members of the research team in this study were clinicians from mental health and addiction services in Girona and also from social services at the time of data recruitment. They were part of a multidisciplinary community team coordinated to address the needs of homeless patients in a comprehensive manner. The patients, before being attended in any of these services, signed an informed consent form in which they accepted the coordination of the professionals of the different services for better social and health care. The professionals of this team, between 2006 and 2020, attended 3,854 homeless people. Thanks to the coordination of health and social services, a shared list of cases with social, mental health, addictions and mortality data was obtained, which identified 391 people who had died during these years.

The research team obtained permission from the *Ethics Committee of the Institut d’Assistència Sanitària* (*CIEC-IAS*), Catalan Institute of Health, with the code Estudi_homeless_2008 on December 12, 2014. And also that of the *Research Ethics Committee CEI GIRONA* that renewed the permission on October 28, 2016 with the project name *Evolució homeless de Girona: Seguiment longitudinal Cohort 2006/2016* (*Code COHORT2006*). As it was not possible to obtain informed consent for research purposes, the Ethics Committee approved the permission for the study in accordance with the exceptions included In articles 7 and 11 of the Organic Law 15/1999 on the Protection of Personal Data. The ethics committee approved the study while this law was in force. In 2018, Organic Law 3/2018, of December 5, on the Protection of Personal Data and Guarantee of Digital Rights, came into force, which modified the previous law authorizing the present study. The new law, in its sixth transitory provision on the reuse for health and biomedical research purposes of personal data collected prior to the entry into force of this organic law states: The reuse for health and biomedical research purposes of personal data lawfully collected prior to the entry into force of this organic law shall be considered lawful and compatible when any of the following circumstances concur: (a) That such personal data are used for the specific purpose for which consent had been given. (b) That, having obtained consent for a specific purpose, such data are used for purposes or areas of research related to the medical or research specialty in which the initial study was scientifically integrated.

The same ethics committee, which is the reference ethics committee of the public health services of the province of Girona, annually renews the permission to continue analyzing the data of the cohort on which this study is based according to the Spanish legality reflected in this text. Therefore, the permission to proceed with research using this database is currently in force, in compliance with Spanish law. The renewal code of the permit is dated January 10, 2023 with the renewal code *COHORT_2016.133*.

Additional Provision 17.2.d LOPD-GDD on pseudo-anonymization of personal data was also followed. To meet this criterion, the research team provided an administrative team with the list of homeless people with their health data (data used in the clinical follow-up of the patients) and the administrative team rearranged the database and assigned a random, anonymous code to each person so that the research team had no record of the person’s identity. The data were then analyzed and used in an aggregated, anonymous and confidential manner.

We included these basic sociodemographic variables: age, gender, marital status, criminal record or prison sentence, and origin. For origin, subjects were identified as Spanish-born or foreign-born, where the former were people born within the national territory of the Spanish state and the latter were people born outside the Spanish state. We define origin is an invariable status that is determined by the individual’s country of birth, regardless of the individual’s citizenship or the immigration status of the individual’s parents or grandparents [24, 25].

We studied variables related to (a) mental health and addiction: having an open medical record in the mental health and addiction network, main diagnosis of substance use disorder, main diagnosis of other mental disorder (eating disorder, attention deficit hyperactivity disorder, autism spectrum disorder, sleep disorder, phobia, behavioral disorder and nonspecific disorder) and dual pathology; (b) clinical diagnosis of infection: hepatitis C virus, HIV and tuberculosis; (c) clinical diagnosis of chronic physical disease: arterial hypertension, type 2 diabetes mellitus (T2D), COPD, cardiovascular disease, obesity, and cancer; and (d) clinical and diagnostic mortality: living/deceased, age at death and cause of death. We composed a list of the people who died during the analysis period and analyzed the variables related to the age at death.

### Statistical analysis

Measures of central tendency and dispersion were used to describe the numerical variables and the absolute and relative frequencies were calculated in the case of categorical variables. We used a Student’s t-test to compare means and a chi-square test to analyze categorical variables. A multiple linear regression model was fitted to the data to establish the predictors of an earlier age at death, after checking the assumption of normality in the distribution of the data (Kolmogorov-Smirnov test), independence of residual values (Durbin-Watson test), collinearity (tolerance and variance inflation factor) and homogeneity of the variance between Spanish-born and foreign-born IEHs (Levene’s test). We included variables related to a lower age at death in the bivariate analysis.

## Results

The cause of death for eight IEHs could not be established, and therefore they were eliminated from the mortality analysis. Thus, the analysis was carried out on 383 people, of which 281 were Spanish-born and 102 foreign-born. The leading cause of death was suicide (n = 92), followed by cirrhosis of the liver (n = 70), overdose (n = 58), COPD (n = 42), heart disease/infarction (n = 36), acquired immune deficiency syndrome (AIDS) (n = 36), cancer (n = 36), meningitis (n = 31) and sepsis (n = 18).

### Variables associated with age at death

The mean age at death of the 383 people was 52.38 years (SD = 13.23; range 22–89). Table 1 shows that the Spanish-born IEHs died younger (M = 48.65 years) than the foreign-born IEHs (M = 57.82 years, t(382) = 1.476, p < .001). People with a criminal record also died earlier than people who had no criminal record (50.87 years vs. 59.00 years, t(124) = -2.655, p = .009).

**Table 1:** Comparison of the mean age at death according to sociodemographics, mental health, infectious diseases, and chronic diseases.

	Age at death M (SD)		Values		
Variables	Presence of the variable	Absence of the variable	*t*	gl	p
Sociodemographics					
Gender (Male)	52.30 (13.58)	52.74 (11.58)	-0.281	114.50	.780
Foreign-born	57.82 (13.67)	48.65 (13.42)	1.476	382.00	< .001
Marital status (married)	52.69 (12.96)	52.88 (13.52)	-0.135	328.84	.892
Criminal record	50.87 (13.16)	59.00 (8.550)	-2.655	124.00	.009
Prison sentence	53.92 (14.87)	51.08 (11.42)	1.133	80.790	.261
Mental health disorders					
Substance use disorder					
Alcohol	51.20 (13.11)	56.80 (12.81)	-3.505	129.90	.001
Opiates	44.87 (10.53)	53.01 (13.25)	-3.277	382.00	.001
Cocaine	44.50 (1.732)	52.46 (13.27)	-1.198	382.00	< .001
Cannabis	53.83 (12.23)	52.91 (11.54)	2.028	92.340	.211
Multiple drug use disorder	52.61 (12.32)	55.94 (16.31)	-1.077	48.250	.287
Other mental disorders					
Psychotic	51.74 (14.98)	52.45 (13.05)	-0.282	43.280	.780
Personality	52.05 (13.11)	57.00 (14.42)	-1.701	28.030	.100
Mood	52.12 (13.24)	56.33 (12.64)	-1.577	26.420	.127
Adaptive	52.28 (13.38)	55.50 (6.510)	-0.830	382.00	.407
Dual pathology	49.18 (10.87)	52.69 (13.41)	-1.759	43.210	.086
Infectious diseases					
Hepatitis C	48.32 (12.42)	52.97 (11.87)	-2.911	382.00	.009
HIV	49.88 (10.22)	53.39 (14.16)	-2.387	382.00	.017
Tuberculosis	49.12 (10.22)	53.91 (14.16)	-2.077	382.00	.021
Chronic diseases					
Arterial hypertension	50.73 (12.32)	61.43 (14.47)	-5.391	75.410	< .001
Obesity	53.87 (10.04)	52.26 (13.50)	0.815	38.390	.420
T2D	51.67 (13.24)	57.42 (12.13)	-3.037	63.810	.003
Cardiovascular disease	51.54 (12.64)	58.12 (15.65)	-2.841	58.780	.006
COPD	51.41 (12.64)	61.32 (15.27)	-4.489	382.00	< .001
Cancer	51.95 (13.31)	56.78 (12.64)	-2.168	43.430	.036

Note: SD = standard deviation, HIV = human immunodeficiency virus, T2D = type 2 diabetes mellitus, COPD = coronary obstructive pulmonary disease

With respect to mental health diagnoses related to substance use disorder, people with alcohol use disorder died earlier (51.20 years vs. 56.80 years, t(129) = -3.505, p = .001), as did people with opiate use disorder (44.87 years vs. 53.01 years, t(382) = -3.277, p = .001) and cocaine use disorder (44.50 years vs. 52.46 years, t(382) = -1.198, p < .001) (see Table 1). No significant differences were found in the age at death for the rest of the mental health disorders, whether related to substance use disorder or not.

In terms of infections, mean differences in the age at death were found in all of infection variables: hepatitis C (48.32 years vs. 52.97 years, t(382)= -2.911, p = .009), HIV (49.88 years vs. 53.39 years, t(382)= -2.9387, p = .017) and tuberculosis (49.12 years vs. 53.91 years, t(382)= -2.077, p = .021) (see Table 1). Finally, regarding chronic organic diseases (see Table 1), differences were found in the age at death in people diagnosed with arterial hypertension (50.73 years vs. 61.43 years, t(75.4)= -5.391, p < .001), T2D (51.67 years vs. 57.42 years, t(63.8) = -3.037, p = .003), cardiovascular disease (51.54 years vs. 58.12 years, t(58.7)= -2.841, p = .006), COPD (51.41 years vs. 61.32 years, t(382) = -4.489, p < .001) and cancer (51.95 years vs. 56.78 years, t(43.4) = -2.168, p = .036).

### Predictors of a lower age at death

Before performing the multiple linear regression, we tested for normal distribution of the variable age at death (Kolmogorov-Smirnov = 1.171, p = .129), independence of the residual values (Durbin-Watson = 1.877), and collinearity (tolerance = 0.894, variance inflation factor = 1.231). The results of the linear regression with deceased persons (see Table 2) showed a link to age at death for COPD (b = − 0.348), not being foreign-born (b = 0.324), cocaine use disorder (b = − 0.169), opiate use disorder (b = − 0.243), cardiovascular disease (b = − 0.223), alcohol use disorder (b = − 0.199), tuberculosis (b = − 0.163), HA (b = − 0.203), criminal record (b = − 0.167) and hepatitis C (b = − 0.129). The variables included in the model explained 89% of the variance in the age at death.

**Table 2:** Multiple linear regression with dependent variable as age at death for IEHs (R2 = 0.895).

	Non-standardized coefficients		Standardized coefficients		
Model	B	SE	Beta	t	Sig.
(Constant)	21.47	5.293		6.513	< 0.001
COPD	-7.714	1.705	-0.348	-5.284	< 0.001
Foreign-born	6.626	5.748	0.324	1.849	0.05
Cocaine use disorder	-6.046	2.930	-0.169	-2.271	0.002
Opiate use disorder	-5.580	1.681	-0.243	-2.915	< 0.001
Cardiovascular	-5.539	1.952	-0.223	-2.349	0.001
Alcohol use disorder	-5.132	1.671	-0.199	-2.669	0.001
Tuberculosis	-4.769	1.845	-0.163	-2.126	0.003
Arterial hypertension	-4.501	1.768	-0.203	-2.112	0.003
Criminal record	-3.687	1.275	-0.167	-2.677	0.001
Hepatitis C	-3.240	1.365	-0.129	-1.373	0.021

Note: SE = standard error, COPD = coronary obstructive pulmonary disease. Variables in the model: COPD, foreign-born, cocaine use disorder, opiate use disorder, cardiovascular, alcohol use disorder, tuberculosis, arterial hypertension, criminal record, hepatitis C, opiate use disorder, multiple drug use disorder, dual pathology, gender, personality disorder, obesity

Given the weight of the Spanish-born/foreign-born variable, two separate linear regression models were fitted to the data to observe the differences in the age at death in relation to the predicting variables, one for the Spanish-born IEH subjects and the other for the foreign-born IEH subjects. Regarding the Spanish-born IEHs (see Table 3), the regression showed the following predictors of the age at death: opiate use disorder (b = − 0.675), COPD (b = − 0.479), cocaine use disorder (b = − 0.208), arterial hypertension (b = − 0.358), multiple drug use disorder (b = − 0.365), cardiovascular disease (b = − 0.306), dual pathology (b = − 0.286), female gender (b = − 0.181), personality disorder (b = − 0.201), obesity (b = − 0.123), tuberculosis (b = − 0.120), and criminal record (b = − 0.153). The variables included in this model explained 87% of the variance in the age at death of Spanish-born people. Regarding the foreign-born IEHs, linear regression showed the following predictors of age at death: psychotic disorder (b = − 0.134), tuberculosis (b = − 0.132), opiate use disorder (b = − 0.119) and alcohol use disorder (b = − 0.098) (see Table 4). In this case, the variables included in the model explained 78% of the variance in the age at death of foreign-born people.

**Table 3:** Multiple linear regression with dependent variable as age at death for Spanish-born IEHs (R2 = 0.869)

	Non-standardized coefficients		Standardized coefficients		
Model	B	SE	Beta	t	Sig.
(Constant)	29.35	3.233		19.07	< 0.001
Opiate use disorder	-9.472	2.300	-0.675	-6.465	< 0.001
COPD	-7.364	1.972	-0.479	-5.282	< 0.001
Cocaine use disorder	-6.195	3.677	-0.208	-5.044	0.004
Arterial hypertension	-6.012	2.578	-0.358	-4.885	< 0.001
Multiple drug use disorder	-5.591	2.478	-0.365	-4.870	< 0.001
Cardiovascular diseases	-4.919	2.277	-0.306	-4.037	< 0.001
Dual pathology	-4.795	1.770	-0.286	-4.177	< 0.001
Gender (female)	-4.493	2.841	-0.181	− .3285	0.027
Personality disorder	-4.326	2.789	-0.201	-2.268	0.028
Obesity	-3.490	2.757	-0.123	-2.991	0.052
Tuberculosis	-3.713	2.283	-0.120	-2.064	0.044
Criminal record	-2.596	1.832	-0.153	-1.509	0.015

Note: SE = standard error, COPD = coronary obstructive pulmonary disease. Variables in the model: COPD, foreign-born, cocaine use disorder, opiate use disorder, cardiovascular, alcohol use disorder, tuberculosis, arterial hypertension, criminal record, hepatitis C, opiate use disorder, multiple drug use disorder, dual pathology, gender, personality disorder, obesity

**Table 4:** Multiple linear regression with dependent variable as age at death for foreign-born IEHs (R2 = 0.781)

	Non-standardized coefficients		Standardized coefficients		
Model	B	SE	Beta	t	Sig.
(Constant)	-10.86	3.484		-2.294	0.045
Psychotic disorder	-3.703	0.111	-0.134	-33.33	< 0.001
Tuberculosis	-3.653	0.103	-0.132	-35.54	< 0.001
Opiate use disorder	-3.291	0.125	-0.119	-26.33	< 0.001
Alcohol use disorder	-2.109	0.146	-0.098	-22.12	< 0.001

Note: SE = standard error, COPD = coronary obstructive pulmonary disease. Variables in the model: COPD, foreign-born, cocaine use disorder, opiate use disorder, cardiovascular, alcohol use disorder, tuberculosis, arterial hypertension, criminal record, hepatitis C, opiate use disorder, multiple drug use disorder, dual pathology, gender, personality disorder, obesity

## Discussion

We analyzed mortality and age at death among IEHs in Spain, comparing Spanish-born individuals and foreign-born individuals. The leading cause of death among the sample was suicide, but cirrhosis, overdose, and COPD were also major causes of death. Notably, one in four deaths was by suicide. Suicide is a serious, global public health issue worldwide and its prevention has been included in the United Nations Sustainable Development Goals [26]. Given that suicide is markedly prevalent among IEHs [27, 28], the reduction of suicide mortality should be a priority in this population. Care should be taken to ensure that suicide prevention programs reach IEHs. In this sense, providing a telephone and Wi-Fi service to IEHs could help them access basic and specialized health services [29, 30], considering the good results of self-harm prevention lines [31] and the existence of this type of program in Spain [32].

Cirrhosis was the second leading cause of death. Several studies show the high prevalence of chronic liver disease in IEHs, compared to the general population, mainly due to chronic hepatitis C infection and alcohol abuse [33]. Hepatitis C is almost five times more prevalent in IEHs. The effectiveness of new treatments has dramatically reduced the incidence and complications of chronic hepatitis C infection in developed countries and today, hepatitis C is concentrated in hard-to-treat populations, such as IEHs. Homelessness or unstable housing hinders the success of hepatitis C treatment. Adherence could be improved by dispensing antivirals in health facilities and observing patients as they take them, either in facilities or on the street (in the latter case, by teams of street social workers/educators) [34].

Another striking finding was that the mean age at death was 52 years. This age is drastically lower than that recorded for the Spanish general population, which is estimated at 83 years [35]. Disorders related to alcohol, opiates, and cocaine use clearly influenced earlier age of death, as did hepatitis C, HIV, tuberculosis, and several chronic conditions (e.g., arterial hypertension, T2D, COPD, cardiovascular disease, etc.). These results are in accordance with previous studies indicating that IEHs experience high rates of morbidity, mortality, and premature death [2, 23, 36, 37].

Concerning predictors of age at death, and in line with other studies, the largest differences in mortality rates were for smoking-related diseases: respiratory diseases and ischemic heart disease [38]. The strongest predictor across the whole sample was the occurrence of COPD. COPD is particularly prevalent among IEH adults, and respiratory tract infections are common in this group [7, 36]. The prevalence of COPD in IEHs is probably underestimated, given the difficulty of these patients to receive complementary tests, such as spirometry, to confirm the diagnosis. The handling and proper use of inhalers is also complex, hampered by the sometimes high price of inhalers and the frequent loss of both medicines and inhalation chambers. IEHs’ poor oral health and missing teeth can cause an improper fit of the chamber and therefore a loss of effectiveness. The use of Beta 2 adrenergic drugs, such as salbutamol, can create substance use disorder in these patients, and tachycardia, produced as a side effect, can worsen previous cardiovascular disease.

The approach to smoking in this population deserves a special attention. In clinical practice, we often find IEHs who have smoked since childhood or adolescence and who consume more than one pack a day. IEHs use the cheapest cigarette brands, which are especially rich in tar and nicotine. They may reuse cigarettes by collecting cigarette butts and use newspaper as rolling paper, thus increasing their exposure to infection and toxins [39]. Tobacco is not the only substance they may consume in this way. Marijuana, smoked or snorted cocaine, and volatile substances also affect the lungs over time [40]. Traditionally, professionals from health and mental health services have tended to tolerate tobacco consumption in IEHs, considering it a lesser evil among the substance use disorders and pathologies they present [41]. In fact, some professionals have been reported to offer IEHs cigarettes as a way of establishing rapport [42]. These actions should be abandoned and, given the high mortality and years of life lost due to smoking in the IEH population, specific programs should be designed to support IEH smokers who want to quit. In fact, there are no differences between IEHs and the general population when it comes to quitting smoking [43], and IEHs can benefit from the usual strategies aimed at smokers in the general population, such as helping them to increase their perception of self-efficacy and their social support [44]. IEHs face substantial barriers to quitting smoking, and there is still insufficient evidence to assess the effects of tobacco cessation interventions in the IEH population [45]. Although tobacco use disorder disproportionately affects disadvantaged groups, they are often underrepresented in medical research and treatments [46]. It is essential to maximize enrollment and retention strategies adapted to IEHs.

The presence of cardiovascular disease was another predictor of death. IEHs have an approximately three times greater risk of cardiovascular disease and an increased cardiovascular mortality than the general population [47]. Several factors contribute to this disparity. In addition to the high prevalence of smoking, there is poor monitoring of cardiovascular risk factors such as hypertension and diabetes. Psychosocial factors such as chronic stress, depression and substance use disorder use confer an additional risk of poor monitoring and adverse effects. Poor access to medical care leads to delays in diagnosis and treatment. Multidisciplinary collaboration between healthcare professionals and social workers is necessary for success in managing IEHs with chronic cardiovascular disease [48].

Additionally, separate regression models for Spanish-born individuals and for foreign-born individuals revealed differential predictors of age at death. For instance, opiate use disorder was the best predictor specifically for Spanish-born individuals, but psychotic disorder and tuberculosis were the best predictors for foreign-born individuals. These results suggest the usefulness of implementing specific prevention programs tailored to the features and needs of each subgroup.

Another important finding was that the mean age at death was nine years earlier for Spanish-born individuals in comparison with foreign-born individuals (49 years vs. 58 years, respectively). Our results are consistent with those of other studies that have revealed that non-immigrant background is a predictor of death among IEHs [37, 49]. Moreover, Spanish-born individuals showed worse overall health, e.g., higher prevalence of substance use disorders, multiple drug use, serious mental health disorders, and several chronic physical illnesses. Notably, health problems and mortality among foreign-born IEHs were also troublingly prevalent, but to a lesser extent compared to Spanish-born individuals.

A possible explanation for these results is the healthy immigrant effect, according to which foreign-born individuals are healthier and live longer than native-born individuals [19]. Likewise, the children of foreign-born parents are healthier and live longer than the children of locally born parents (although the outcomes vary depending on the mother’s country of origin) [21]. Our results suggest that this effect holds for the IEH population. As noted by Oliva-Arocas et al. [50], there may be protective cultural elements whereby the immigrant population would maintain more favorable mortality outcomes due to healthier habits related to diet or substance use. Specifically, it would be interesting to study why COPD affects foreigners less. Could it be due to lower tobacco consumption?

This hypothesis would fit with our data, as alcohol and drug use, obesity, hypertension, and respiratory diseases were substantially less prevalent among foreign-born individuals. However, the literature is not consistent on the existence of this healthy immigrant effect and both methodological aspects as well as certain variables may modulate the effect [19, 51]. In this line, a study in the Spanish general population found that mortality was lower in foreign-born individuals, compared with Spanish-born individuals [24]. However, nationality, country of origin, and length of stay in the host country influenced this effect. For instance, no significant differences in mortality were found when the length of stay in Spain was over 10 years.

Regardless of the healthy immigrant effect, the causes of death among foreign and native IEHs are different, as are the reasons why they reach a state of literal homelessness. These differences may have implications for the provision of services, highlighting the need to develop health and social care programs adapted to newcomers, in order to avoid more risk factors associated with homelessness and the concomitant deterioration of health [10]. Policies to protect the unemployed from poverty, increase employability and restore employment opportunities are needed to prevent the deterioration of the health of the population. The immigrant population must be protected by such policies, given that this group is especially vulnerable to the effects of economic and social crises [20].

Our results are subject to certain limitations. First, our sample was not designed to be representative of all IEHs in Spain, which could limit the generalizability of our results. Second, foreign-born IEHs may have migrated to other regions of Spain or other European countries, thus disappearing from Girona’s records. Third, this study only included data from people living literally homeless; unfortunately, it does not consider mortality among people who experience other forms of homelessness and housing exclusion. Lastly, we classified all foreigners in the same category, using country of birth as a segregation variable, without considering other variables related to the migration process, such as length of residence, administrative status, or belonging to an ethnic minority. Future research should take these elements into account in order to outline more precisely the variables of migration related to mortality and health.

Notwithstanding these limitations, the study suggests an alarming pattern of morbidity, mortality, and premature death among IEHs in Spain, especially among Spanish-born individuals. Our results show that most deaths were due to medical causes, especially cirrhosis, COPD, cardiovascular disease, and AIDS. Future studies should consider tobacco use in IEHs. While taken as a group, medical causes were the most prevalent, certain external causes of death (suicide, overdose) should be highlighted as they accounted for almost 40 per cent of overall deaths. Urgent efforts are needed to address the complex healthcare needs of IEHs and to reduce morbidity and mortality rates in this population.

## Data Availability

Complete raw data are not publicly available to preserve participants’ anonymity in line with the obtained ethical approval for this study. However, further de-identifed data can be made available upon reasonable request from the corresponding author.
